# Species identification of ivory and bone museum objects using minimally invasive proteomics

**DOI:** 10.1126/sciadv.adi9028

**Published:** 2024-01-26

**Authors:** Catherine Gilbert, Vaclav Krupicka, Francesca Galluzzi, Aleksandra Popowich, Katell Bathany, Stéphane Claverol, Julie Arslanoglu, Caroline Tokarski

**Affiliations:** ^1^ University of Bordeaux, CNRS, Bordeaux INP, CBMN, UMR 5248, F-33600 Pessac, France.; ^2^ Bordeaux Proteome Platform, University of Bordeaux, F-33000 Bordeaux, France.; ^3^ Department of Scientific Research, The Metropolitan Museum of Art, New York, NY, USA.

## Abstract

Ivory is a highly prized material in many cultures since it can be carved into intricate designs and have a highly polished surface. Due to its popularity, the animals from which ivory can be sourced are under threat of extinction. Identification of ivory species is not only important for CITES compliance, it can also provide information about the context in which a work was created. Here, we have developed a minimally invasive workflow to remove minimal amounts of material from precious objects and, using high-resolution mass spectrometry–based proteomics, identified the taxonomy of ivory and bone objects from The Metropolitan Museum of Art collection dating from as early as 4000 B.C. We built a proteomic database of underrepresented species based on exemplars from the American Museum of Natural History, and proposed alternative data analysis workflows for samples containing inconsistently preserved organic material. This application demonstrates extensive ivory species identification using proteomics to unlock sequence uncertainties, e.g., Leu/Ile discrimination.

## Teaser

Minimally invasive proteomics is used for rapid conversion of ancient bone and ivory samples into species-specific taxonomies.

## INTRODUCTION

Ivory has been used as a carving material in many cultures in part because of its numerous aesthetic qualities; it has a very fine grain, polishes well, and has a warm texture and color (*1*, *2*). Hence, ivory is a material commonly found in museum collections, in the form of statuettes, buttons, chess pieces, and configured into a variety of spiritual and household objects as inlays or individual components (furniture legs, stair newels, etc.). Strictly speaking, the term ivory refers to material derived from tusks, that is, teeth that grow continuously throughout an animal’s life, such as those of elephants. However, the term is also more widely used to describe any teeth that have been carved, such as those of hippopotamus and sperm whales (*3*, *4*).

Ivory taxonomic identification is of great interest not only for museums (for the identification of materials and movement of cultural heritage objects around the world) but also for the regulation of the trade or trafficking of materials from endangered species. Several species from which ivory can be sourced are protected under the Convention on International Trade in Endangered Species of Wild Fauna and Flora (CITES), and thus, an accurate species identification of seized objects is vital to prosecutors. CITES is also relevant when artworks are purchased or requested for loan from museums, which involves conservators as well as curators and registrars/collections managers. When species cannot be determined by visual methods (*1*, *5*), museums often find full characterization of an object nearly impossible.

A common question for wildlife law enforcement officers is the distinction between mammoth ivory and elephant ivory, as while the trade of mammoth ivory is unregulated, the trade of elephant ivory is prohibited under CITES, and is thus frequently passed as mammoth ivory. In addition to this, in 2020, there was a notable increase in hippopotamus teeth being seized at European Union borders, caused by the increased restrictions in the trade of elephant ivory, with calls now starting for hippopotamus to be added to the list of CITES-protected species (*6*). While customs officials today use visual identification based on the identification materials produced by governmental departments (e.g., the United States Fish and Wildlife Department), the development of analytical-based techniques represents a major support and also improves the conservation effort (*7*–*9*). The World Wildlife Foundation’s “*Identification Guide for Ivory and Ivory Substitutes*” cites a variety of analytical procedures for ivory species identification, including ultraviolet spectroscopy, microscopic analysis of Schreger lines, and DNA analysis, but there is an absence of data and studies to show the strength of proteomics for this application (*10*). The identification of elephant ivory using DNA analysis is becoming a vital tool in prosecutions relating to hunting and poaching, where methods typically require 200 mg of material (*11*, *12*). DNA analysis has also been used to identify the species of origin of museum objects, including an elephant ivory chess piece, and sperm whale teeth, where tens of milligrams of material were required to confidently identify the species of origin (*13*, *14*). Other nondestructive methods such as infrared and Raman spectroscopies are also used to classify species-specific features (*15*, *16*).

As mentioned above, ivory is essentially an elongated tooth, and therefore, the majority of the material is formed of enamel and dentin. Ivory is composed of a thin outer layer of cementum, an enamel derivative, and a main core of dentin (*17*). One should note that, even if enamel was present initially, it is often worn away during the lifetime of the animal (e.g., the elephant has enamel only at the tip of the tusk, but it is usually not present in the mature elephant’s tusk; in addition, even when present, the enamel is usually removed during carving). Dentin has a higher organic content than enamel of around 20% by weight, of which 90% are collagenous proteins, predominantly collagen type I (*18*). These collagenous proteins form mineralized collagen fibrils throughout the dentinal matrix, the structure of which is very ordered, made up of a series of tubules that align themselves into sheets of microlaminae (*17*). The orientation of these microlaminae sheets relative to each other produces characteristic Schreger lines, which are commonly used to differentiate proboscidean (elephant and mammoth) ivories (*17*).

Nondestructive methods such as x-ray diffractometry have been used to discriminate between mineralized tissues, such as bone, antler, and ivory (*19*), whereas small and wide-angle x-ray scattering, cross-polarized light microscopy, and scanning electron microscopy have all been used to study orientation and three-dimensional (3D) arrangement of the mineralized collagen fibers in ivory that relate to the macroscopic Schreger pattern (*20*, *21*). In addition to this, synchrotron radiation micro–computed tomography (SR-μCT) scanning has been used to characterize the 3D morphology of ivory, antler, land mammal, and whale bones at a high resolution to differentiate them (*22*). SR-μCT has also been used to study the 3D microtubule network to explain the appearance of the Schreger pattern (*23*). The structure of the inorganic matrix of ivory can be studied using particle-induced x-ray emission (micro-PIXE) analysis to better understand the conservation state of an object (*24*, *25*). Micro-PIXE can also be used to study trace metal compositions, which can allow for the distinction between ivory and bone objects, as well as the identification of the geographical origin of ivory objects, and the state of ivory preservation based on the fluorine content and MgO/CaO ratios (*26*–*31*).

Destructive analysis can yield more specific or conclusive information regarding the age and taxonomy of a particular object, as such techniques can have a far higher specificity than nondestructive, spectroscopy-based techniques. Of course, in such cases, a balance must be found between sampling enough material to have a conclusive result and not compromising the integrity of the object. For example, stable isotope analysis (^13^C, ^15^N, and ^86^Sr) of historic samples has been used to track the ivory trade throughout Africa, although such analyses typically require up to 50 mg of sample (*32*–*35*). Another destructive technique commonly used for species identification is peptide mass fingerprinting (PMF), also referred to as Zooarchaeology by mass spectrometry (ZooMS) when applied to collagenous tissues (*36*). ZooMS has proven to be an excellent tool for screening large numbers of samples quickly during large-scale archaeological investigations (*37*). However, when a limited amount of sample is available, a clear result is not always guaranteed. Liquid chromatography coupled to mass spectrometry (LC-MS)–based proteomics has higher sensitivity and accuracy. For PMF, protein identification depends on measurements of peptide molecular weights (MS spectra), whereas with the LC-MS method, peptides are separated by LC and additionally fragmented in the analyzer of the mass spectrometer, allowing for accurate amino acid sequences elucidated from the fragment ions in the peptide fragmentation spectra (MS/MS spectra). This technique has also successfully been used in archaeological investigations for species identification of morphologically unidentifiable bone fragments, both in the mainstream data-dependent acquisition, and, more recently, in a high-throughput data-independent acquisition (*38*–*40*).

In this work, we present the use of minimally invasive LC-MS/MS–based proteomics used to perform taxonomic identification of ivory and bone museum objects. This methodology was applied on a highly diverse group of objects based on their age, preservation state, and suspected species of origin from The Metropolitan Museum of Art’s collections: Egyptian Art, Medieval Art, Musical Instruments, and The Michael C. Rockefeller Wing, which includes sub-Saharan Africa, the Pacific Islands, and North, Central, and South America collections. In addition to the analysis of museum objects, elephant tusks and hippopotamus teeth and bones collected in the early 1900s from the Mammalogy Department of the American Museum of Natural History (AMNH) were sampled to construct a homemade proteomic database of underrepresented species. Furthermore, we developed targeted data analysis methodologies to extract information from highly degraded historical objects. Last, we developed MS3 fragmentation methods enabling the differentiation of leucine and isoleucine isomeric residues for more confident sequence identification. From the protein extracted from a minimal sample amount (i.e., small amount of powdered bone or ivory), we show here the successful identification of a variety of species from the studied objects based on the identification of multiple unique peptides that were fully fragmented across the peptide backbone.

## RESULTS

While proteomics was originally developed for biological applications, in recent years, it has been increasingly used to study museum and archaeological objects. Typically, proteomic analysis is used either to perform accurate identification of proteinaceous material, taxonomic identification, and phylogenetic analysis, or to better understand preservation of ancient samples (*40*–*43*). A key benefit to using proteomics in comparison to DNA sequencing for such analysis is that, generally, proteins persist longer in mineralized tissues than DNA (*44*–*46*).

Collagens, particularly type I, are the most prevalent structural proteins in mineralized tissues such as bones and ivory, and therefore, the taxonomic identification detailed in this study is based on peptides identified from these proteins. However, the collagen sequence is highly conserved between species, and between closely related species, very few amino acid substitutions are present. In addition to this, many of the species from which ivory originates have not had their genome fully sequenced, and there is little information available on public proteomic/genomic databases. For these reasons, to increase the likelihood of identifying taxonomically informative peptides, the sample preparation and analysis must be optimized to target collagen and maximize its coverage. In this study, a total of 16 objects in the Metropolitan Museum of Art collections were analyzed varying in their age, from ~4000 to 800 years old, provenance, and in their preservation condition (Table 1 and table S1). Table 2 shows the sequence coverage and the number of collagen peptides detected for each sample where a taxonomic assignment was possible.

**Table 1.** Samples analyzed in this research where a taxonomic assignment was possible, including accession number, description of the objects, sample analyzed, sampling location, notes on condition or treatment, and taxonomic identification.

	MET accession number	Title	Sample	Location description	Treatment/condition notes	Identification		
						Family	Genus	Species
*Samples from The Metropolitan Museum of Art*								
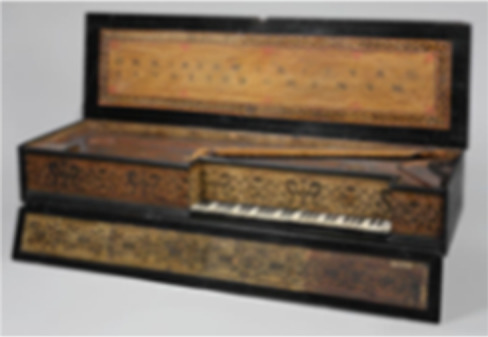	11.176.1	Muselar Virginal by J. Ruckers	Second and third rubbings	S16, D5 key	*–*		*Bos*	
			Second and third rubbings	S17, A4 key	–		*Bos*	
			Second and third rubbings	S18, B2 key	–		*Bos*	
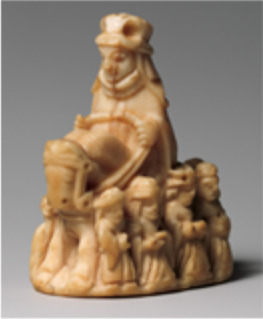	2012.346	Queen chess piece, Scandinavia, 13th century	Second rubbing	Base of figure at rear	–			*P. macrocephalus*
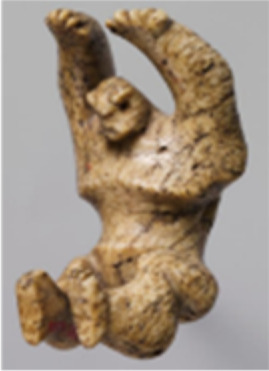	1979.206.1587	Pendant, Hawaii, 18th to 19th century	Fourth rubbing	Inside of hole	–			*P. macrocephalus*
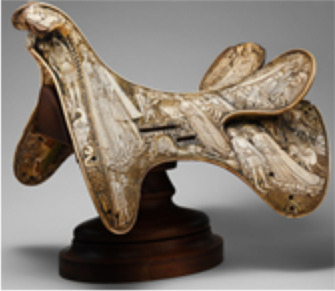	40.66.a,b	Saddle, Central Europe, approximately 1400–1420	Second rubbing	S1, Decorative trim	*–*	Cervidae		
			Second rubbing	S2, body of the saddle	*–*		*Bos*	
			Second rubbing	S3, decorative trim (another sampling location)	*–*	Cervidae		
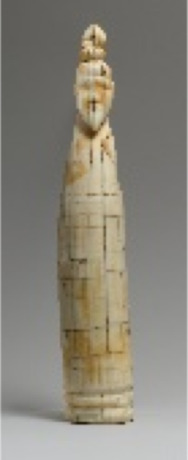	66.99.1	Tusk figure of a man, Egypt, approximately 3900–3500 B.C.	Second rubbing	Reverse rim, interior surface	Good condition with glossy surface			*H. amphibius*
	26.7.1284	Furniture leg fragment, Egypt, approximately 2960–2649 B.C.	Second rubbing	Lower edge opposite of perforations, lighter area within break edge	Lighter area appears to be a recent break			*H. amphibius*
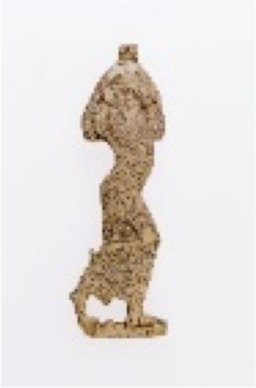	66.99.50	Figure of an Asiatic captive, Egypt, approximately 1295–1070 B.C.	Second and third rubbings	Reverse of waist, along break area	Break previously joined		Bos*	
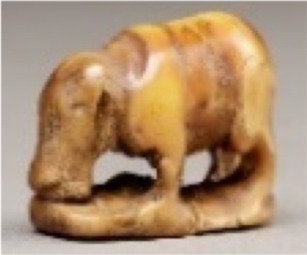	26.7.1299	Figurine or amulet of a hippo on a sled, Egypt, approximately 2960–2649 B.C.	First rubbing	Ground below loss of proper right front leg	Darkened surface with potential coating			*H. amphibius*
	12.187.30	Spoon, approximately 3100–2649 B.C.	Second rubbing	Bottom edge, below perforation	Previously consolidated with Paraloid B-72		Bos*	
	**Mammalogy AMNH accession number**	**Title**	**Sample**	**Location description**	**Treatment/condition notes**	**Identification**		
						**Family**	**Genus**	**Species**
*Samples from the American Museum of Natural History*								
-	51949	Democratic Republic of Congo, Upper Uele, Yakuluku, 3 October 1911 (collected by H. Lang and J. Chapin)	Fourth rubbing	Base of tusk	-			*L. africana*
-	51950	Democratic Republic of Congo, Upper Uele, Yakuluku, 3 October 1911 (collected by H. Lang and J. Chapin)	Fourth rubbing	Base of tusk	-			*L. africana*
-	52094	Democratic Republic of Congo, Upper Uele, Faradje, 1 December 1911, (collected by H. Lang and J. Chapin)	Fourth rubbing	Base of tusk	-			*L. africana*
-	113816	Africa, Fan Awaing, 4 September 1938 (collected by W. D. Campbell)	Fourth rubbing	Inside pulp cavity	-			*H. amphibius*
-	90302	Zoo, collected 28 April 1929	Fourth rubbing	Inside pulp cavity	-			*H. amphibius*
-	81856	Collected in Africa, no collection date	Fourth rubbing	Inside pulp cavity	-			*H. amphibius*

*Possible previous consolidation with bovine glue.

**Table 2.** Sequence coverage and the number of peptide identifications for objects where a taxonomic assignment was possible.

Accession number	Description	Identification			Collagen α1(I)		Collagen α2(I)		# Taxon-specific peptides identified
		Family	Genus	Species	% Coverage	# Peptides	% Coverage	# Peptides	
11.176.1	Virginal D5 key		Bos*		70	993	73	705	53
	Virginal A4 key		Bos*		71	1060	72	751	92
	Virginal B2 key		Bos*		70	837	71	620	76
1979.206.1587	Pendant			*P. macrocephalus*	69	593	66	429	68
2012.346	Queen chess piece			*P. macrocephalus*	63	259	56	227	66
40.66.a,b	Saddle S1	Cervidae*			62	213	54	172	16
	Saddle S2		Bos*		67	493	66	382	85
	Saddle S3	Cervidae*			70	740	69	506	30
66.99.1	Tusk figure of a man, approximately 3900–3500 B.C.			*H. amphibius*	92	534	78	325	111
66.99.50	Figure of an Asiatic captive, approximately 1295–1070 B.C.		Bos*		68	474	69	362	67
26.7.1284	Furniture leg fragment, approximately 2960–2649 B.C.			*H. amphibius*	68	132	51	89	68
26.7.1299	Figurine or amulet of a hippo on a sled, approximately 2960–2649 B.C.			*H. amphibius*	59	101	51	82	14†
12.187.30	Spoon, approximately 3100–2649 B.C.		Bos*		55	79	46	60	17†

*Identification was only possible at the genus level due to the high conservation of the collagen sequence between species within *Bos* genus and Cervidae family, respectively.

†Identification confirmed using peptide mass fingerprints and retention time of the peptides.

### Taxonomic analysis of ancient collagens from represented species in referenced databases

#### Minimally invasive analysis

The development of protocols for proteomics that require minimal amounts of sample material can enable access to precious museum and archaeological collections, as the sampling has a negligible impact on the integrity of the object. To perform successful minimally invasive analysis, it is necessary to miniaturize and optimize both the sample preparation and data acquisition methods. Various sampling techniques have been proposed to limit the effect that the sampling can have on the object, such as the use of polyvinyl chloride–free erasers and microgrit films (*47*, *48*). Other sampling techniques that have been proposed include enzyme functionalized films and sampling strips designed for clinical use (*49*, *50*). It is important to note that the type of sampling performed must be designed and optimized for the specific material that will be sampled. Furthermore, to minimize the sample loss during protein extraction, steps can be taken such as integrating different chemical treatments into a single or few steps, or using miniaturized analytical workflows, both of which have already demonstrated to improve recovery in the most challenging ancient samples (*47*, *51*).

In this analysis, we used a highly effective minimally invasive sampling technique using 6-μm diamond microgrit polishing films (*47*, *52*). During sampling, where possible, three to four sequential rubbings were taken from an object, performing the sampling at the same location each time. Each of these samples was visible as a small amount of powdered material on the surface of the microgrit film, pictured in fig. S1. This sequential sampling technique allowed for the removal of the topmost layer of contamination and also access to potentially better preserved material underneath the external surface.

Investigations of this sampling technique using exemplar archaeological teeth showed that performing at least one rubbing before taking the sample that will be analyzed substantially increases the sequence coverage of collagen, from around 30% coverage of collagen α1(i) and α(ii) in the first samples, to 70% of coverage for both in the second sampling. The effects of this sampling technique are summarized in table S2.

A key strength of the protocol used in this study is the evaporation of the trifluoroacetic acid (TFA)–based demineralization solution, as this allowed for all the material extracted from the samples to be analyzed in one injection. This means that the proteins solubilized by the demineralization step, which would have usually been analyzed as a different fraction or discarded, were combined with the acid-insoluble fraction. To further maximize the protein recovery, the extraction, reduction, and alkylation steps were performed simultaneously, to reduce sample loss caused by additional reaction steps. The benefits of this are demonstrated by the high sequence coverage and number of peptide identifications not only in the exemplar samples but also in the museum objects.

#### Represented species: Single-component studies

**Fig. 1.**
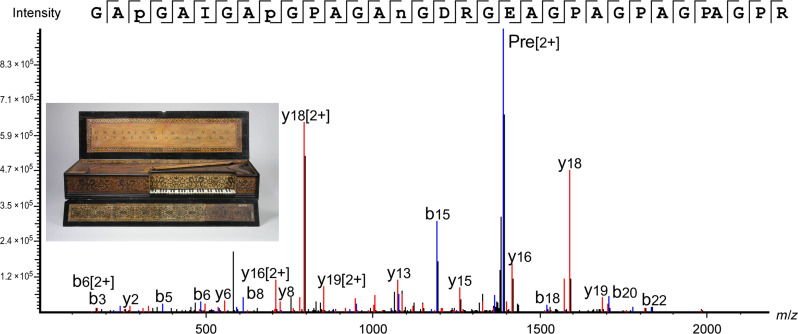
*Bos* peptide marker identified in Muselar Virginal sample—11.176.1, J. Ruckers, 1622, The Metropolitan Museum of Art. Peptide from collagen α2(i), indicative of *Bos* genus identified in all three samples taken from object 11.176.1. GAP(+15.99)GAIGAP(+15.99)GPAGAN(+0.98)GDRGEAGPAGPAGPAGPR, *m*/*z*: 1391.1682, *z*: 2, parts per million (ppm): 1.2.

In the following examples, we present three objects that are made from a single piece of bone or ivory that originate from species whose collagen sequences are represented in public databases. In the first case, we applied the minimally invasive method described above to samples taken from the polished white keys of the Muselar Virginal (11.176.1, J. Ruckers, 1622). The resulting data showed an excellent sequence coverage of more than 70% for collagens α1(I) and α1(I) (Table 2). All three samples taken from the different keys were found to be made from bone from the *Bos* genus. An example is shown in Fig. 1 of a peptide that was detected across all three samples taken, which is specific to the *Bos* genus, and the extinct species *Toxodon*, highly unlikely given the context. Details of the number of collagen peptides detected can be found in Table 2, and a full list of the Bovinae peptides identified across the three samples, including those specific to *Bos*, can be found in table S3. This case study benefited from the high sequence coverage of collagen identified using our minimally invasive method along with available sequence data in the public databases covering the *Bos* genus and related species.

**Fig. 2.**
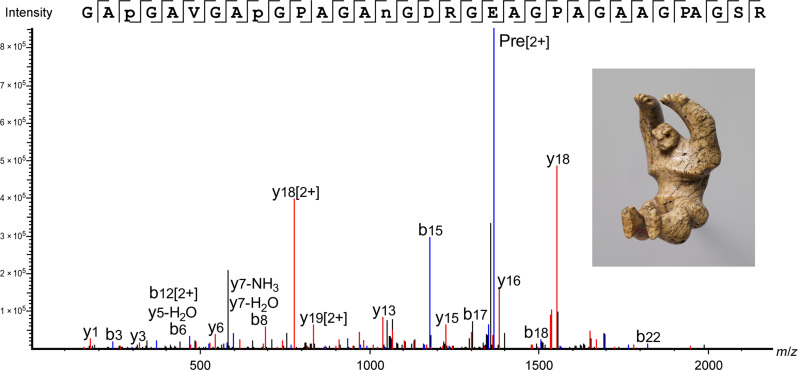
Sperm whale peptide marker identified in Hawaii bone pendant sample—1979.206.1587, 18th to 19th century, The Metropolitan Museum of Art. Peptide from collagen α2(i), unique to *P. macrocephalus*, detected in object 1979.206.1587, but also identified in object 2012.346. GAP(+15.99)GAVGAP(+15.99)GPAGAN(+0.98)GDRGEAGPAGAAGPAGSR, *m*/*z*: 1366.1423, *z*: 2, parts per million (ppm): 0.1.

In the next cases, a 13th-century Scandinavian ivory queen chess piece (2012.346) and an 18th- to 19th-century Hawaii bone pendant (1979.206.1587) were identified as sperm whale (*Physeter macrocephalus*), based on the identification of 66 and 68 species-specific peptides, respectively (see tables S4 and S5). Unique parts of the collagen sequence were detected in the form of multiple peptides with different modifications, as well as missed and unspecific cleavages, with spectra that were fully fragmented across the peptide backbone, as shown in Fig. 2. One should note that the peptides shown in Figs. 1 and 2 are relatively long, and both include missed tryptic cleavages. While the same peptide sequences with no missed cleavages were also identified, the spectra of the longer peptides were of better quality and had higher identification scores. After the initial search with an untargeted database containing all the collagen sequences in UniProt including whale collagens, a targeted database search was performed using the Odontoceti parvorder in the National Center for Biotechnology Information (NCBI), which is made up of toothed whales. This was performed as *P. microcephalus* is closely related to many other species of whale, including narwhal (*Monodon monoceros*), another source of ivory. It should be noted that this identification of *P. microcephalus* was made on the basis of the information available on online databases and that of the 138 species part of the Odontoceti parvorder, only 33 species have collagen sequenced (importantly *M. monoceros* is sequenced and not identified here). Therefore, while this was a confident species assignment based on collagen sequences currently available, the species identification may be refined as public databases are updated.

#### Represented species: Multicomponent studies

**Fig. 3.**
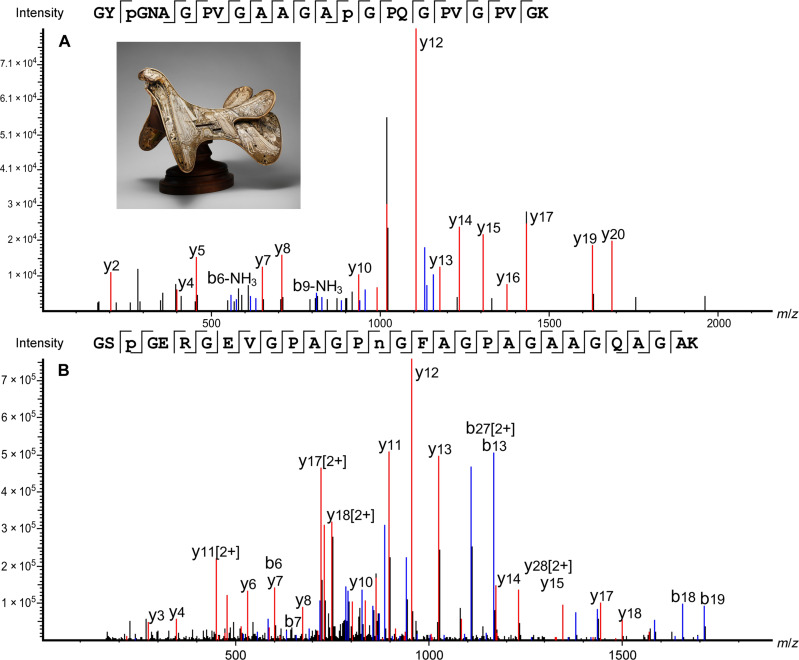
Taxon marker peptides identified in ceremonial medieval saddle sample—40.66.a,b, approximately 1400–1420, The Metropolitan Museum of Art. (**A**) Peptide from collagen α2(i) unique to the Bovinae subfamily, detected in sample S2 from object 40.66a,b GYP(+15.99)GNAGPVGAAGAP(+15.99)GPQGPVGPVGK, *m*/*z*: 1131.0693, *z*: 2, parts per million (ppm): 2.5. (**B**) Peptide from collagen α2(i) used in conjunction with others (see tables S7 and S8) to identify the Cervidae family detected in samples S1 and S3 from object 40.66a,b GSP(+15.99)GERGEVGPAGPN(+0.98)GFAGPAGAAGQAGAK, *m*/*z*: 870.4167, *z*: 3, ppm: 2.3.

Other assignments were more difficult because of the complexity of the object construction and preservation history. For example, it was challenging to assign a taxonomy to a ceremonial medieval saddle decorated with bone plaques (40.66.a,b, approximately 1400–1420) because, unlike the other objects in this study, the saddle is an assemblage of different materials, rather than a carving of a single piece of bone or ivory. It was believed to be made from antler, bone, raw hide, and bone-based glue from various animals, including deer and cows. This complicated the analysis as there was a possibility that samples could contain a mixture of collagen from different species. For this object, three samples from three different locations were analyzed, detailed in Table 1. Of the three samples, one was concluded to be from the *Bos* genus, and two from the Cervidae family, but it was not possible to perform a more specific taxonomic assignment due to the high conservation of the collagen sequences between species in both the *Bos* genus and Cervidae family. For Cervidae, the availability of data in the current databases was also a limitation (of the 251 descendants that make up the Cervidae family, only 6 have collagen sequenced on the NCBI database). Following the untargeted database search, a more targeted search was performed using a database from the infraorder Pecora (an infraorder of even toed hoofed mammals, including Bovinae and Cervidae). For the body of the saddle (S2), 85 peptides unique to the Bovinae subfamily were identified, an example is shown in Fig. 3A, and a list of the peptides identified is shown in table S6.

For the samples taken from the decorative trim (S1 and S3 from object 40.66a,b), both were concluded to contain collagen from the Cervidae family. The collagen α2(i) sequence between residues 707 and 736, which is specific to both the Cervidae and Elephantidae families was identified in both samples (Fig. 3B). However, the collagen α2(i) sequence showed differences in other regions, and in conjunction to confidently differentiate between Elephantidae and Cervidae: The collagen α2(i) sequence between residues 947 and 972 is unique to the Cervidae family. Samples S1 and S3 were therefore both concluded to contain collagen from Cervidae, based on the identification of 16 and 30 informative peptides, respectively, as listed in tables S7 and S8.

### Taxonomic analysis of collagens from underrepresented species in databases

#### Creation of databases of underrepresented species

As discussed above, the samples analyzed in this study were very diverse, and some were particularly challenging due to the suspected species of origin of the ivory. For example, ivory originating from Egypt is typically elephant (*Loxodonta africana*) tusks or hippopotamus (*Hippopotamus amphibius*) teeth (*53*). These two species have not been well-studied using proteomics, and there is limited information available on public proteomic and genomic databases. The collagen protein sequences present in public databases are either fragments (*H. amphibius*) or reconstructed from DNA sequences (*L. africana*) (*54*, *55*). Considering this lack of reference information, we built our own proteomic database for the species *L. africana* and *H. amphibius*. Samples were taken from the Mammalian collections of the American Museum of Natural History using the same minimally invasive sampling method applied to the museum objects. The information related to the pieces from which the objects were taken is available in Table 1.

**Fig. 4.**
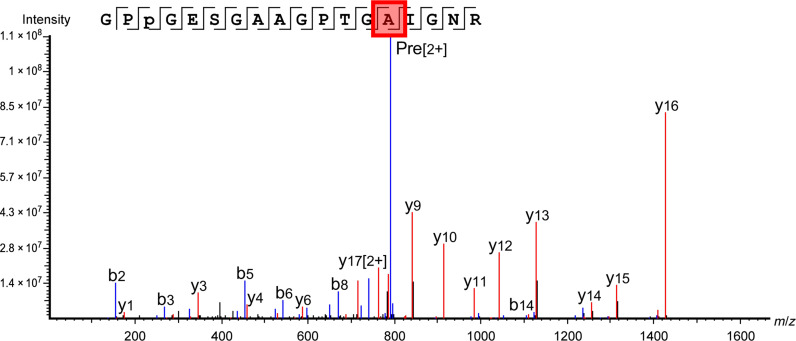
*H. amphibius* marker peptide identified in exemplar sample, collected in Africa by W. D. Campbell, 4 September 1938—113816, The American Museum of National History. MS/MS spectrum showing the identification of the substitution P → A at position #455 in collagen α2(i) peptide from *H. amphibius* in exemplar 113816, Mammalogy collection, AMNH. GPP(+15.99)GESGAAGPTGAIGNR, *m*/*z*: 791.3815, *z*: 2, parts per million (ppm): 1.6.

Using these exemplars, we were not only able to validate the species identification of the museum objects but also identify residues in the collagen sequences that did not align with those in the public databases. For example, residue #455 in collagen α2(i) of *H. amphibius* is reported to be P in the public databases, but multiple peptides were identified where residue #455 is A. Table S9 shows an increase in the number of peptides identified for all the exemplar samples of *H. amphibius* with an additional 30 collagen α2(i) peptides detected, when incorporating this substitution for the database search. This was confirmed through the identification of the same peptide sequence with different modifications, missed, or unspecific cleavages in all three exemplar samples (table S10), as well as all historical samples that were identified to originate from *H, amphibius*; an example spectrum is shown in Fig. 4. This is an important identification as this peptide sequence is unique to *H. amphibius*, and when searching the data with a classical database search with P at residue #455, this part of the sequence was not identified, while when searching with A in this position, this portion of the sequence was covered by a high number of peptides, as demonstrated in table S10. This substitution was identified both in all three exemplar samples and in all historical samples that were identified to originate from *H. amphibius* (see details in the following sections). One should note that, since the submission of this manuscript, collagen sequences for *H. amphibius kiboko* have been added to the NCBI database, derived from genomic sequences, where A was also referenced (NCBI reference sequence: XP_057587072.1).

In addition to the exemplar samples from *H. amphibius*, three samples from *L. africana* (Savannah elephant) ivory were analyzed to identify unique peptide markers that could be used to validate the species identification of the historical samples. A detailed study of the collagen sequence of *L. africana* was required to search the data from the historical samples. In particular, for collagen α1(i), there are inconsistencies between the protein sequences available on the two databases UniProt and NCBI, as shown in the sequence realignment in fig. S2. Therefore, we were able to use the data collected from the exemplar *L. africana* samples to confirm the collagen α1(i) sequence and confirm it across all three exemplar samples, as detailed in tables S11 and S12. In particular, a G residue was identified at position 326 of collagen α1(i) (table S11), and residues 548 to 551 of collagen α1(i) were confirmed to be GPAG (table S12). With these validated sequences, very high sequence coverages and peptide identifications were identified for the exemplar samples; for sample 51949, 1293 and 991 collagen α1(i) and α1(ii) peptides were detected with a coverage of 73 and 76%, respectively; more details are available in table S9.

#### MS3 method to discriminate leucine/isoleucine

A final hurdle in the challenge of identifying the taxonomy of a sample using LC-MS/MS–based proteomics is the presence of amino acids that have the same mass. When confirming a species-specific peptide, the same peptide but with the isobaric amino acid must also be controlled for, e.g., leucine (L)/isoleucine (I), which have the same mass but two different chemical structures. When trying to investigate historical objects where we expected to identify either *H. amphibius* or *L. africana* ivory, one challenge stemmed from the fact that the publicly available collagen sequences for *H. amphibius* has a sequence uncertainty at every leucine or isoleucine residue (*54*). When comparing the collagen sequences of *H. amphibius* and *L. africana*, many of the identified differences between the sequences are between I and L (illustrated in fig. S3). Therefore, we have developed an MS3 fragmentation method to distinguish between them allowing for a higher confidence species identification. It should be noted that all the data discussed originate from samples taken using the minimally invasive sampling procedure.

Electron-transfer dissociation (ETD fragmentation) was used in MS2, and higher-energy collisional dissociation (HCD fragmentation) was used in MS3 targeting the *z* ions from the MS2 fragmentation. This double fragmentation causes a radical rearrangement that results in a characteristic loss of either 29 Da (isoleucine) or 43 Da (leucine) (*56*, *57*). Using this fragmentation pathway, targeted and untargeted methods were tested. Both methods were successful, but it was found that the targeted method allowed for a more conclusive assignment. Figure S4 shows some examples of spectra that were observed when using an untargeted method, and the informative peaks are at a very low intensity. In the targeted method, several selected peptides that contained either I or L were targeted to unequivocally confirm the amino acid identification. The method developed targeted specific regions of interest of the collagen sequences of both *H. amphibius* and *L. africana*. Through the use of a targeted loss trigger, the signal-to-noise ratio of the MS3 spectra was greatly improved without losing sensitivity for the MS2 fragmentation in case the expected rearrangement reaction in MS3 does not take place.

Figures S5 and S6 show spectra from Mammalogy AMNH objects 52094 (*L. africana*) and 113816 (*H. amphibius*). Figure S5 shows the fragmentation of the peptide sequence containing the substitution at position 456 in collagen α2(i) of *H. amphibius*, to confirm that the I/L in position 456 is indeed isoleucine, thereby fully confirming the amino acid sequence of this section of the collagen α2(i) identified to be highly specific to *H. amphibius*. Figure S6 shows the confirmation of leucine at position 515 of collagen α2(i) of *L. africana*.

#### Underrepresented species: Single-component archaeological studies

The samples from objects originating from Egypt presented more challenges than the others referenced in Table 1 for several reasons: the level of preservation due to the age of the objects (3900–1070 B.C.) or its environment, the species that were expected to be identified, and potential past conservation history that may be undocumented. These objects are precious and fragile archaeological objects and may have been treated when they were first collected. For three objects, species identification was performed using MS/MS spectra and were expected to contain species that are underrepresented in databases, such as *L. africana* and *H. amphibius*. Therefore, using the proteomic data collected from the Mammalogy AMNH exemplar samples, as well as the protein sequences deduced from this data, a confident species assignment was reached for several objects. The Tusk figure of a man (66.99.1, Egypt, approximately 3900–3500 B.C.), the Furniture leg fragment (26.7.1284, Egypt, approximately 2960–2649 B.C.), and the Figurine or amulet of a hippo on a sled (26.7.1299, Egypt, approximately 2960–2649 B.C.) were all identified to be made of ivory from *H. amphibius*. The sequence coverage of collagen α1(i) and α2(i) is shown in Table 2.

**Fig. 5.**
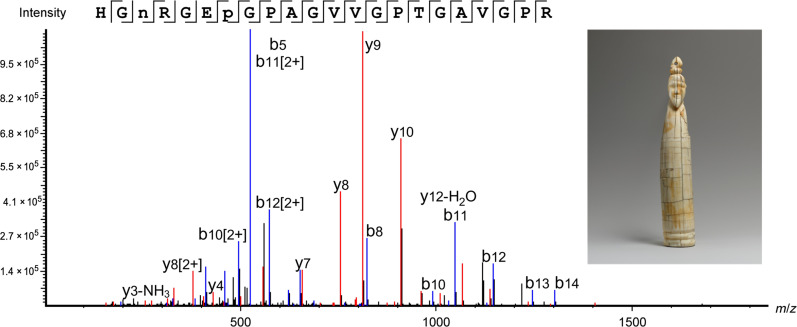
*H. amphibius* marker peptide identified in the Tusk figure of a man sample, 66.99.1, Egypt, approximately 3900–3500 B.C., The Metropolitan Museum of Art. Peptide from collagen α2(i) unique to *H. amphibius*, identified in object 66.99.1 HGN(+0.98)RGEP(+15.99)GPAGVVGPTGAVGPR, *m*/*z*: 686.3525, *z*: 3, parts per million (ppm): 3.3.

The Tusk figure of a man (66.99.1, Egypt, approximately 3900–3500 B.C.) had a good result, with a total of 859 collagen type I peptides detected. Of these, 111 peptides were identified to be specific to *H. amphibius* (see table S13), including peptides containing the substitution at residue #455 from P to A. The peptides identified as unique were typically in the same parts of the collagen sequence but with varying modifications such as deamidation and proline hydroxylation, as well as unspecific and missed cleavages, as demonstrated in table S13. This demonstrates the high level of protein heterogeneity in the samples analyzed, due to both the intrinsic, highly modified nature of collagen and the increase of modifications in ancient proteins due to aging and degradation. One peptide in particular, HGN(+0.98)RGEP(+15.99)GPAGVVGPTGAVGPR [mass/charge ratio (*m*/*z*): 686.0184, *z*: 3, shown in Fig. 5], was identified as a confident marker of collagen from *H. amphibius*, as this peptide has two amino acid substitutions compared to this region of the collagen α2(I) sequence of *L. africana.* It should be noted that this peptide contains a deamidated asparagine (N), which is chemically equivalent to aspartic acid (D); therefore, the same sequence was also searched against the NCBI database with N substituted for D, to validate that the peptide was unique to *H. amphibius*.

**Fig. 6.**
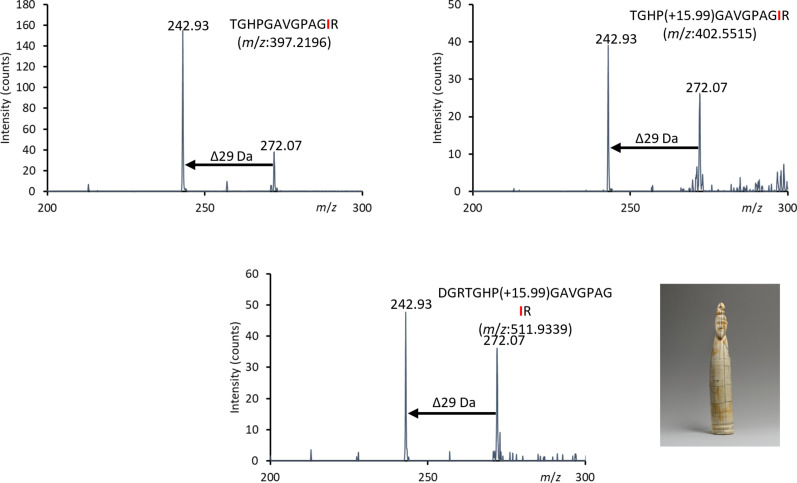
Identification of isoleucine in *H. amphibius* marker peptide in the Tusk figure of a man sample, 66.99.1, Egypt, approximately 3900–3500 B.C., The Metropolitan Museum of Art. MS3 spectra to confirm isoleucine at position #865 of collagen α2(i) of *H. amphibius*, identified in object 66.99.1. Residue identity was confirmed with three different peptides with the same sequence, through the fragmentation of the same *z_2_* ion.

The MS3 method was applied to historic samples to distinguish between leucine and isoleucine. Figure 6 shows the use of this method applied to sample 66.99.1, where a characteristic loss of 29 Da, indicative of isoleucine, is observed for three peptides corresponding to the same collagen sequence with differing modifications and cleavages. Such experiments can serve as an additional tool to identify markers of specific peptides that are indicative of a certain species when such peptide markers contain leucine or isoleucine residues.

The Furniture leg fragment (26.7.1284, Egypt, approximately 2960–2649 B.C.) had lower collagen recovery than the Tusk figure of a man (66.99.1, Egypt, approximately 3900–3500 B.C.), but we still detected 68 and 50% of the collagen α1(i) and α2(i) proteins, respectively. The species-specific peptides of sample 26.7.1284 are shown in table S14, which includes the peptide HGN(+0.98)RGEP(+15.99)GPAGVVGPTGAVGPR, identified in sample 66.99.1 and the exemplar samples as a marker for collagen from *H. amphibius*.

The species of origin of the Figure of an Asiatic captive (66.99.50, Egypt, approximately 1295–1070 B.C.) was visually characterized as ivory (expected to be *H. amphibius*); however, collagen from the *Bos* genus was determined on the basis of the identification of 67 peptides, detailed in table S15. A split in this piece had been treated with Paraloid B-72 in 1985, but the possibility of previous consolidation with bovine glue cannot be ruled out. In particular, collagen Q deamidation profile [a potential marker of time (*58*)] of the 66.99.50 sample does seem to not fit the expected trend for a sample of that age compared with the other samples of this study (see fig. S8). However, a more in-depth investigation on a larger set of samples from studied periods of time and also sample replicates would be beneficial to confirm this trend.

### Taxonomic identification based on LC-MS PMF

**Fig. 7.**
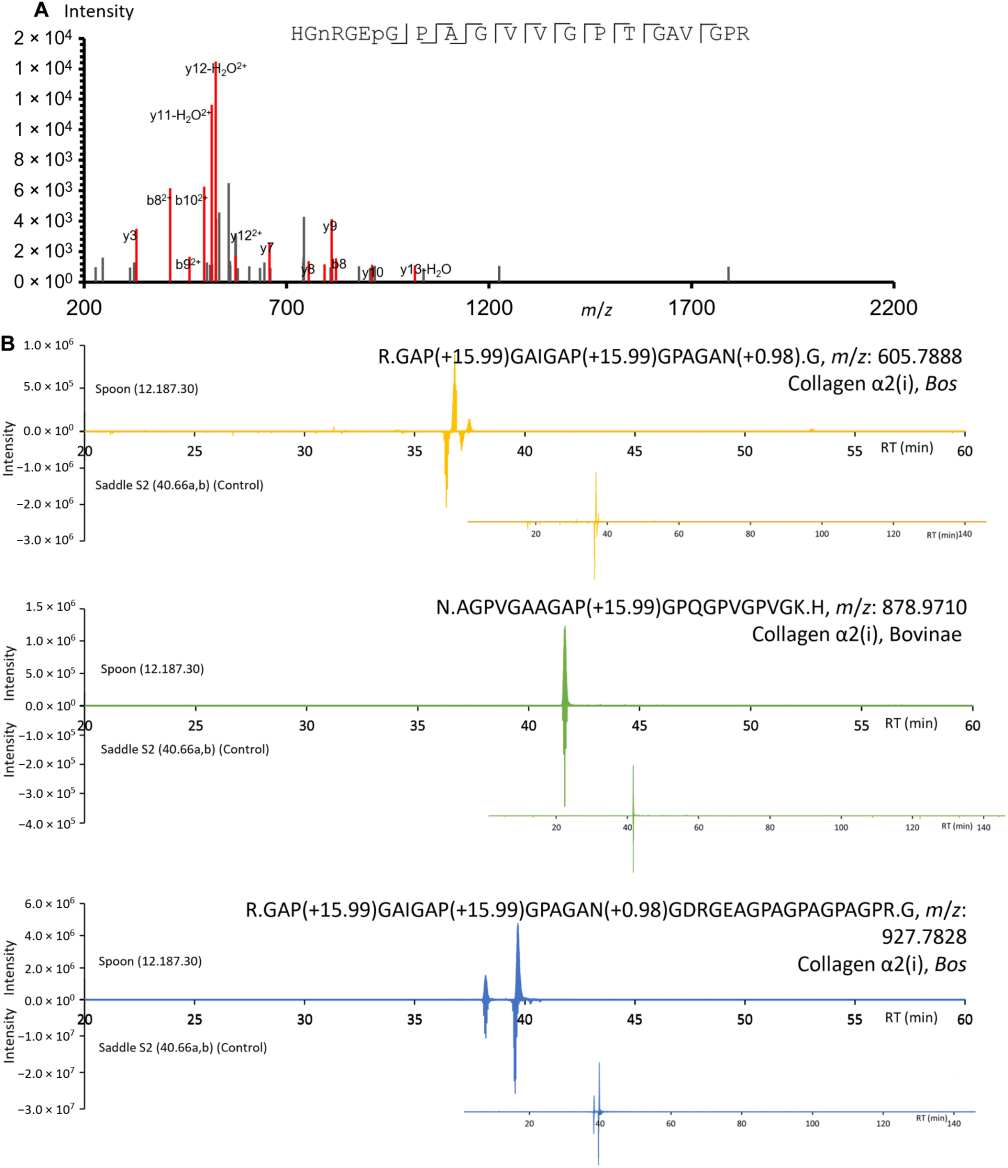
Strategies for identifying species when a sample has poor collagen preservation. (**A**) Peptide from collagen α2(i), HGN(+0.98)RGEP(+15.99)GPAGVVGPTGAVGPR [*m*/*z*: 514.7659, *z*: 4, parts per million (ppm): 1.1], specific to *H. amphibius*, detected in object 26.7.1299; the spectrum that resulted from summing three individual MS2 spectra is shown. (**B**) LC-MS PMF mapping of peptides unique to the *Bos* genus and the subfamily Bovinae identified in sample 12.187.30 and sample 40;66a,b-S2.

For a number of the objects, it was not possible to identify the species, genus, or family from which the ivory originated (table S1). Only collagen peptides were detected, but with a signal-to-noise ratio that was too low to confidently confirm the peptide sequence. The lack of the complete knowledge of an object’s lifetime prevents us from hypothesizing too much on the causes of the poorly preserved organic material. For example, the sample Figurine or amulet of a hippo on a sled (26.7.1299, Egypt, approximately 2960–2649 B.C.) was identified to be *H. amphibius*, but had very poor collagen recovery. Because of the small size of the object (2.5 cm/1 inch) and its fragile nature, only one rubbing was possible. As we previously demonstrated, a reduced number of collagen peptides are detected in the first rubbing from an object. With this low collagen recovery, only 11 peptides unique to hippopotamus were identified during the preliminary database search (see table S16), with poor-quality MS/MS spectra. To extract the maximum amount of information, we used two data processing approaches to more confidently perform the species identification: (i) First, by summing MS/MS spectra of a targeted peptide marker to increase the signal-to-noise ratio to more confidently confirm the amino acid sequence of the peptide (see Fig. 7A) here, both the precursor ion mass and a few fragment ions confirming the sequence are considered for identification; and (ii) second, with the availability of the proteomic data from exemplar samples of *H. amphibius*, we used a peptide mass fingerprint approach (PMF is based only on precursor ions without sequence composition information), but with the additional dimensions of the retention time and charge state (LC-MS PMF) to confirm the presence of informative peptide peaks. The peptide-based approach applied to sample 26.7.1299 is shown in table S17, where the peptides unique to *H. amphibius* were detected both in sample 26.7.1299 and in exemplar sample 113816, one of the exemplar samples that were analyzed during the same sequence. The identification of multiple peptides based on the observed *m/z* values, peptide charge state, and retention time alone allowed for a more confident species assignment of *H. amphibius.*

The low intensity of the collagen peptide peaks caused similar difficulties for the Spoon (12.187.30; approximately 3100–2649 B.C.), where several uninformative collagen peptides were detected that were fully fragmented along the peptide backbone (an example is shown in fig. S7), but a confident taxonomic assignment based on the fragmentation spectra was not possible. Therefore, the LC-MS–based PMF approach was applied, where the *m*/*z* values of several taxon-specific peptides that were identified in other samples (exemplar and historic), including *H. amphibius* and *L. africana*, were searched in the data from the Spoon (12.187.30; approximately 3100–2649 B.C.). In this case, peaks corresponding to peptides specific to the *Bos* genus and Bovinae subfamily were identified, with *m*/*z* values, charge states, and retention times identical to those identified in other samples such as the Medieval Saddle (40.66a,b-S2, approximately 1400–1420). It should be noted that the extracted ion chromatograms of the informative peptides can contain multiple peaks, which most likely corresponds to the same peptide with the same number of modifications but with the modification on a different position on the peptide (e.g., hydroxyproline and deamidation). Three such comparisons of extracted ion chromatograms (EICs) are shown in Fig. 7B, and others are listed in table S18. It was concluded that sample 12.187.30 contained collagen from the *Bos* genus. The Spoon (12.187.30; approximately 3100–2649 B.C.) was also visually characterized as ivory (*H. amphibius* or *L. africana*), and the *Bos* identification raises the possibility of previous treatment with bovine glue (see Q deamidation profile; fig. S8), although there is no record of such a treatment.

## DISCUSSION

We present here a proteomics workflow adapted to the analysis of ancient proteins from an extremely small amount of material. The successful analysis was possible by the optimization of the whole analytical workflow from the miniaturized and simplified sample preparation procedure to the optimized data acquisition and data processing steps. This study is also the first example of the use of LC-MS/MS–based proteomics to perform identification of a variety of species of ivory objects in a museum collection. Materials originating from hippopotamus, elephant, sperm whale, deer, and cow were identified using these combined techniques. We were able to attribute a taxon with a high level of confidence to objects where the species of origin was previously unknown, based on the detection of multiple unique peptide sequences.

To achieve these results, another challenge to take into consideration for the differentiation between species or phylogenetic analysis was the high collagen sequence homology. Depending on the species from which the material originates, the type of object, the availability of sequence information, and the preservation state of the object, we have shown that a species identification can be reached with different levels of targeted data interrogation. For some objects and species that are of particular significance to cultural heritage but had been previously understudied using proteomics techniques, a more in-depth investigation into the collagen sequence itself was required, particularly enriching the existing databases by the study of specimens in natural history collections. Our developments have pointed out several inconsistencies in the current public databases (e.g., for *H. amphibius* and *L. africana*), providing more specific information for species discrimination. Furthermore, we have proposed the use of two consecutive stages of mass spectrometric fragmentation to discriminate between leucine and isoleucine, a methodology never proposed before applied to cultural heritage. In the future, as sequence reconstruction using proteomics becomes more prevalent, such a methodology can allow for the discrimination between leucine and isoleucine residues without the need to refer to DNA data. For samples where organic material was too poorly preserved to confidently assign a species based on a classical database search, we have proposed LC-MS PMF mapping as an alternative taxonomic identification technique. Several pieces of information originating from the LC-MS data were successfully used including the *m*/*z*, the retention time, and the peptide charge state.

Regarding the studied objects, this minimally invasive protocol allowed the unequivocal identification of animal species for ivory objects, which has a high impact on the analysis of cultural heritage objects where sampling is initially not possible. Many of the objects sampled in this study would previously not have had analysis permitted due to the amount of sample required. Objects from the Department of Egyptian Art were of particular interest because of the specific cultural connotations associated with the use of hippopotamus or elephant ivory. In ancient Egypt, hippopotami were both feared and respected as dangerous creatures, often depicted in tombs showing their hunting by kings, symbolizing the royals’ triumph over chaos and maintenance of order (*59*, *60*). Elsewhere, Taweret, a goddess who protected women and young children in ancient Egyptian religion, took the composite form incorporating a hippopotamus, crocodile, lion, and pregnant woman, making her a fierce protector (*59*, *60*). While hippopotami have not inhabited Egypt since the 19th century, the species was common during antiquity and often hunted because of its symbolism, as well as its threat to domestic crops and danger to boats and people around the Nile River (*61*). During the same period, elephants were not as prevalent in northern Africa, although their ivory was still a highly valued material that was traded from southern Africa (*62*). The type of ivory, either elephant or hippopotamus, used to craft an object can be either an indication of the availability of raw materials or the symbolism in the ancient Egyptian culture. The figurine of a hippo on a sledge (26.7.1299, Egypt, approximately 2960–2649 B.C.) being made of actual hippopotamus ivory may serve to bridge materiality and symbolism (*59*). The overwhelming dominance of hippopotamus ivory in the objects studied may simply speak to the availability of species in ancient Egypt (*59*, *60*).

In relation to the other objects included in this study, identifying the taxonomy of the ivory or bone provides substantial information regarding material sourcing for several cultures and an insight into the process of creating the object. For example, the medieval saddle (40.66a,b, Central European, approximately 1400–1420), was constructed from an assemblage of materials: a large ornately carved bone from *Bos* genus, likely a pelvic bone given its size, was used for the body of the saddle, while small pieces of bone or antler from Cervidae was used to create a decorative trim. The different materials were possibly used because of their physical properties, most notably the size of the single *Bos* bone (*63*). This saddle is even more complex in manufacture, being assembled materials. The species identification of ivory objects can also provide context and insight related to the societies from which they originate, such the queen chess piece made of whale ivory (2021.364, 13th century), originating from Scandinavia, a region that has a history of nautical exploration, including whale hunting (*64*). Another example is the Hawai’ian pendant (1979.206.1587, United States, Hawai’i, 18th to 19th century), which was also identified to be made from whale ivory, reflecting the nautical history of Hawai’i (*65*).

The Figure of an Asiatic captive (66.99.50, Egypt, approximately 1295–1070 B.C.) and the Spoon (12.187.30, Egypt, approximately 3100–2649 B.C.) highlight one of the greatest challenges of analyzing art that has a long history of intervention. Both objects were visually characterized as ivory (hippopotamus or elephant) based on visual assessment and yet were identified as *Bos* from proteomic analysis. This suggests contamination from animal glue from a previous undocumented treatment or that these objects are made from cow bone. The latter scenario is of particular interest in that the objects do not have the expected morphological features of bone from an archaeological context. In addition, the deamidation profiles could further indicate that the *Bos* identification is from a perhaps modern consolidant. The methods developed here will become part of the Metropolitan Museum of Art’s routine scientific analysis and will be extended to the international cultural heritage community allowing better characterization and attribution of objects when desired and deemed appropriate by the collections, providing substantial information on the ivory trade, distribution, and use across cultures. On the basis of the demonstrated capabilities of the methods, we also anticipate the use of this workflow in the context of ivory-related prosecutions and seizures.

## MATERIALS AND METHODS

### Samples

The objects analyzed in this study are detailed in Table 1 and table S1. Twenty-two samples from 16 objects from the Metropolitan Museum of Art were analyzed. In addition to this, six samples of ivory originating from *H. amphibius* and *L. africana* were analyzed from the Department of Mammalogy at the American Museum of Natural History. The human archaeological tooth and bone used respectively to test the minimally invasive sampling procedure and as control for MS analysis were obtained from the laboratory "De la Préhistoire à l'Actuel: Culture, Environnement et Anthropologie" (PACEA), UMR CNRS 5199, University of Bordeaux, France.

### Sample preparation

Samples were taken from collections at the Metropolitan Museum of Art and Mammalogy–American Museum of Natural History. Sampling was performed in areas that already displayed a flaw or damage or areas that are not directly visible when the object is on display. There is little to no visible disruption of the surface appearance. Sampling, where possible, was performed by rubbing three to four different pieces of 6-μm diamond microgrit film (Precision Fiber Products) successively over the same area of the surface of the object, removing an immeasurably small amount of material. The successive rubbings were labeled 1, 2, 3, and 4. Table 1 and table S1 show the rubbing that was analyzed for each sample. During the preparation of samples, a microgrit film that had been used to sample a human archaeological bone was used as a control standard, and a protocol blank was prepared and analyzed alongside the samples.

Unless otherwise stated, all reagents were obtained from Merck (Darmstadt, Germany).

The samples were prepared for proteomic analysis using a filter-aided digestion protocol, optimized for minimal sample amounts, adapted from (*66*). First, demineralization was performed by placing each microgrit in an Eppendorf tube and adding 200 μl of 5% TFA. Samples were demineralized for 30 min, at which point the microgrit was removed using tweezers, and the TFA-based solution was evaporated in a vacuum centrifuge until dry. Following this, extraction, reduction, and alkylation were performed in a single step, whereby the samples were incubated in 200 μl of extraction buffer at 60°C for 1 hour in the dark. The extraction buffer consisted of 6 M guanidinium hydrochloride, 60 mM tris-hydrochloride, 5 mM tris(2-carboxyethyl)phosphine, and 15 mM chloroacetamide.

Following the extraction step, the digestion was performed by transferring the extraction solution into a 10-kDa Amicron filter (Merck, Darmstadt, Germany). A buffer exchange was performed by washing each sample three times with a digestion buffer [50 mM ammonium bicarbonate (pH 8.8)], after which 100 μl of digestion buffer was added to the filter. The collection tube for the filters was replaced, 10 μl of trypsin (0.01 μg/μl; Promega, Madison, USA) was then added to each filter, and parafilm was wrapped around the top of each tube to prevent evaporation during the digestion. Digestion was performed overnight (14 hours) at 37°C, in the dark.

To recover the peptides after digestion, samples were centrifuged at 13.2*g* for 10 min, Fifty microliters of digestion buffer was then added to the filters, and the samples were centrifuged for another 10 min (this step was performed three times in total). At this point, the filtrate (containing the digested peptides) was transferred to another Eppendorf tube, acidified with 2 μl of TFA. An extraction with ethyl acetate was then performed, by adding 200 μl of ethyl acetate, aspirating several times with the pipette to mix fully. An additional 800 μl of ethyl acetate was then added to the samples, which were then vortexed, and centrifuged for another 10 min at 13.2*g*. The top, organic, phase was then removed, and the extraction with 800 μl of ethyl acetate was performed two more times. After this, samples were heated at 60°C for 5 min to remove residual ethyl acetate, and then were evaporated to dryness in a vacuum centrifuge. Once dry, 100 μl of 1:1 water:methanol was added, and the samples were evaporated to dryness again, to remove residual volatile salts. Samples were then reconstituted with 2 μl of 0.1% formic acid for analysis.

### MS analysis

#### LC-MS/MS analysis

Samples were analyzed for using a Dionex Ultimate 3000RSLCnano system coupled to an Orbitrap Eclipse mass spectrometer (Thermo Fisher Scientific). Both settings with and without preconcentration column were evaluated as follows, but it was confirmed that the setting without precolumn allowed for a higher sensitivity. One microliter of sample was injected onto a C18 Pepmap Neo trap column (Thermo Fisher Scientific), which was then washed in backflush mode before being injected onto a PepMap RSLC C18 column (2 μm, 100 Å, 75 μm by 50 cm; Thermo Fisher Scientific), with a gradient of 146 min and a flow rate of 300 nl/min. As mentioned earlier, tests were also performed without trap column. Where buffer A was 0.1% formic acid in water and buffer B was 20% water/80% acetonitrile with 0.1% formic acid, the following gradient was used: 0 to 3 min, 4% B; 3 to 96 min, increase to 40% B; 69 to 121 min, increase to 90% B; 121 to 125 min, hold at 90% B; 125 to 126 min, decrease to 4% B; and 126 to 146 min, hold at 4% B. Peptides were detected using an Orbitrap Eclipse mass spectrometer in positive mode. Both MS1 and MS2 spectra were collected in the Orbitrap analyzer, with a mass range of 375 to 1500 and 120,000 resolving power for MS1 and a mass range of 50 to 2000 and 30,000 resolving power for MS2. MS2 spectra were acquired using a data-dependent analysis method, including the following MS2 filters: MIPS (peptide), charge state (2 to 7), and dynamic exclusion (30 s), with a 100% AGC target. MS2 fragmentation was performed with HCD at 28%, using the automated injection time mode.

During analysis, a tryptic digest of human archaeological bone was analyzed at the beginning and end of the sequence to monitor the performance of the instrument during analysis. In addition to this, a minimum of two blanks were placed between every sample to avoid carryover between the runs, and before samples were injected, the data from the blank runs were searched with PEAKS to confirm that there was no carryover.

#### MS3 method

To distinguish between leucine and isoleucine residues, both targeted and untargeted methods were tested. These tests were developed using the same Dionex Ultimate 3000RSLCnano system coupled to an Orbitrap Eclipse mass spectrometer as discussed above, with the same 146-min gradient. For the targeted tests, peptides of interest containing leucine or isoleucine residues were identified, and *m*/*z* values with a charge state of more that 3+ were selected using the targeted mass trigger followed by the targeted mass inclusion filters available on Xcalibur (version 4.2, Thermo Fisher Scientific, Massachusetts, USA). MS2 fragmentation was performed using ETD, with an accumulation time fixed at 300 ms, using the calibrated charge-dependent ETD parameters. *z* ions corresponding to the fragment beside the leucine/isoleucine residue were then targeted using the mass trigger and inclusion filters and fragmented using low-energy HCD fragmentation (12%). A targeted loss trigger was then included so that if a loss of either 29 or 43 Da was detected, three more MS3 spectra would be collected. One hundred percent AGC target and 200-ms injection time were used for MS3. The data were then manually analyzed to identify the characteristic loss of either 43 or 29 Da, indicative of leucine or isoleucine, and spectra originating from the same *z*-ion fragment were averaged to reduce the signal-to-noise ratio.

For the untargeted tests, a method was adapted from (*67*). In short, for each ion that is fragmented, two MS2 spectra are recorded, one using HCD fragmentation (stepped collision energy, 25, 35, and 50%) and the second using EThcD fragmentation (27% supplemental activation). For the analysis of the peptide sequence, the scans using HCD fragmentation are more informative, but to distinguish between leucine and isoleucine, the scan using electron-transfer/higher-energy collision dissociation (EThcD fragmentation) is used. To perform the differentiation, data were searched manually, for characteristic w ions that have a loss of either 29 or 43 Da, which can indicate the residue at a certain position.

#### Data processing

Proteomic data analysis was performed with PEAKS X (Bioinformatic Solutions, Waterloo, Canada). Multiple interrogations were performed on each dataset. The first interrogation used was performed using a database containing all the collagen sequences in the UniProt database (downloaded 16 June 2022), while the second interrogation was performed with a targeted database containing the sequences of species known to produce ivory from the NCBI database (downloaded 2 March 2022, Odontoceti database downloaded 3 April 2022, and Pecora downloaded 8 March 2022). The cRAP (common Repository of Adventitious Proteins) database was used as a contaminant database in all the searches to control for contamination. The search was performed with a parent mass error tolerance of 10.0 parts per million (ppm) and a fragment ion error tolerance of 0.02 Da. The enzyme used in the search was trypsin, with a semispecific cleavage and a maximum of three missed cleavages. Carbamidomethylation was set as a fixed modification, while deamidation (NQ) and oxidation (MP) were set as variable modifications. A false discovery rate of 0.1% was used, with five variable modifications allowed. Peptides identified as species specific through the targeted database search were then manually controlled by searching the peptide sequence using the NCBI BLAST database search tool. The COBALT algorithm, also available on NCBI, was used when performing sequence alignments to identify informative regions of the sequence. If a species-specific peptide contained leucine or isoleucine, the same peptide sequence with the respective amino acid was searched, and only considered a species-specific peptide if no other species were hits for the second search. For manual species identification, data visualization was performed in Xcalibur version 4.2. MS spectra were summed with the QtiPlot program using multipeak fit setting and extracted ASCII-encoded data from MS raw files. The PTM profile tool available in PEAKS X was used to extract the Q deamidation data from raw files. PEAKS records the intensity of modified PSMs with the detected signature ions as well as the intensity of unmodified PSMs, where the analog signature ions were detected. PTM profile provides the abundance of modified and unmodified forms containing the PTM sites identified, which was used to produce the frequency plot of detected signature ions for Q deamidation.
