# Supplementary material for: Species identification of ivory and bone museum objects using minimally invasive proteomics
Source: Sci Adv. 2024 Jan 26;10(4):eadi9028. doi: 10.1126/sciadv.adi9028 (PMC10816696; doi:10.1126/sciadv.adi9028)
Supplement: Supplementary file 1 — Figs. S1 to S8 Legends for tables S1 to S18 [file sciadv.adi9028_sm.pdf]

Science Advances
AAAS

Supplementary Materials for

Species identification of ivory and bone museum objects using minimally invasive proteomics

Catherine Gilbert *et al.*

Corresponding author: Caroline Tokarski, caroline.tokarski@u-bordeaux.fr

Sci. Adv. **10**, eadi9028 (2024)
DOI: 10.1126/sciadv.adi9028

The PDF file includes:

Figs. S1 to S8
Legends for tables S1 to S18

Other Supplementary Material for this manuscript includes the following:

Tables S1 to S18

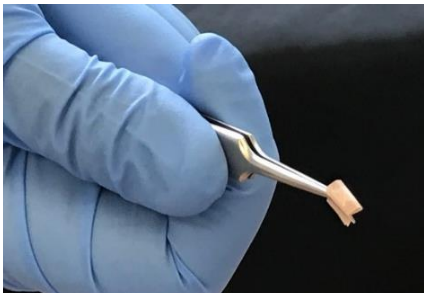

Fig. S1.

The samples received for analysis. Pictured is the 6 µm diamond polishing film with a small amount of dust collected from the surface of the object.

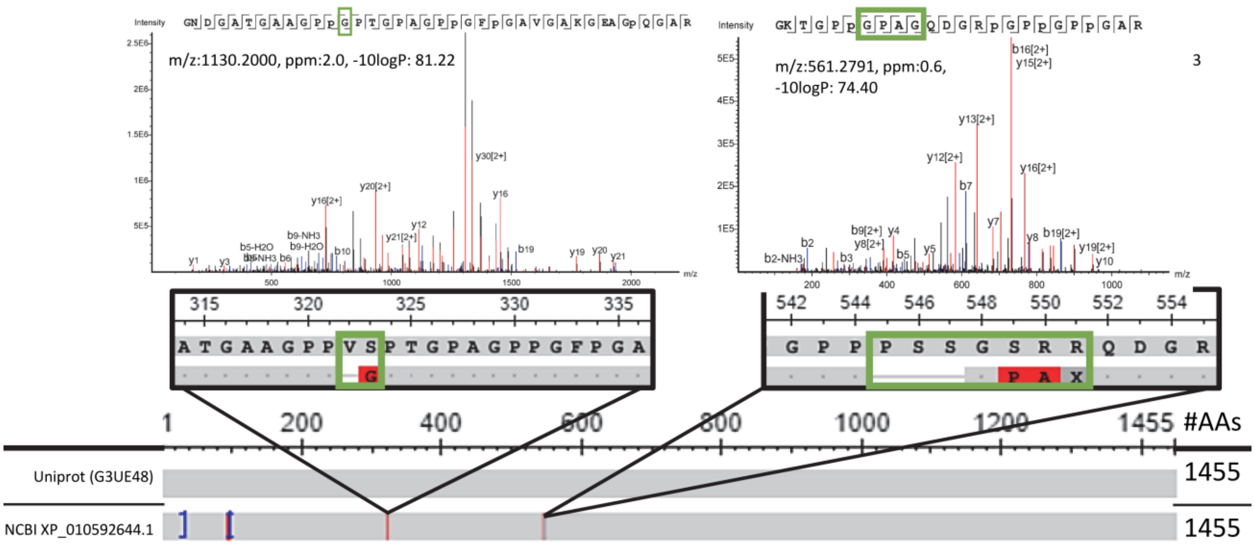

Fig. S2.

Alignment of the collagen α1(I) sequences of *Loxodonta africana* available on the NCBI and Uniprot protein databases, with MS/MS spectra showing the identification of the accurate sequence based on the data from the exemplar samples.

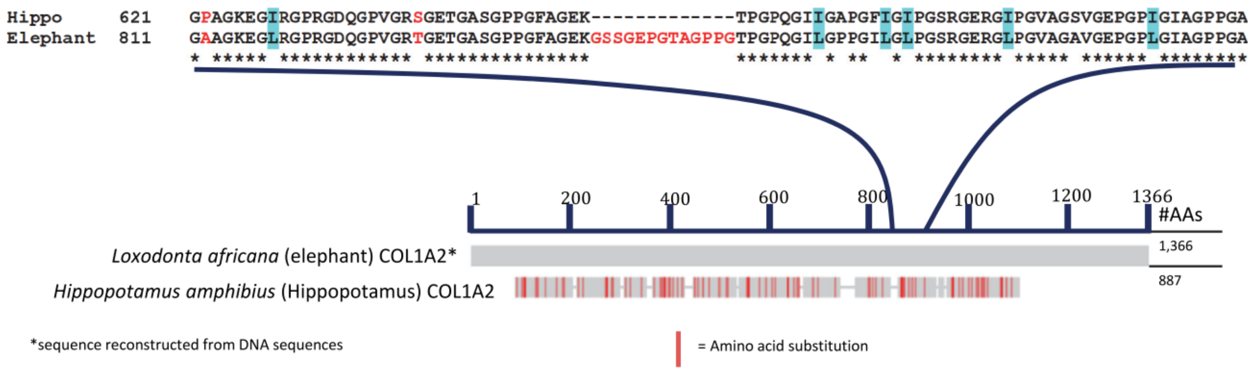

Fig. S3.

Most of the identified substitutions between the collagen α2(I) of *Hippopotamus amphibius* and *Loxodonta africana*, are between leucine and isoleucine.

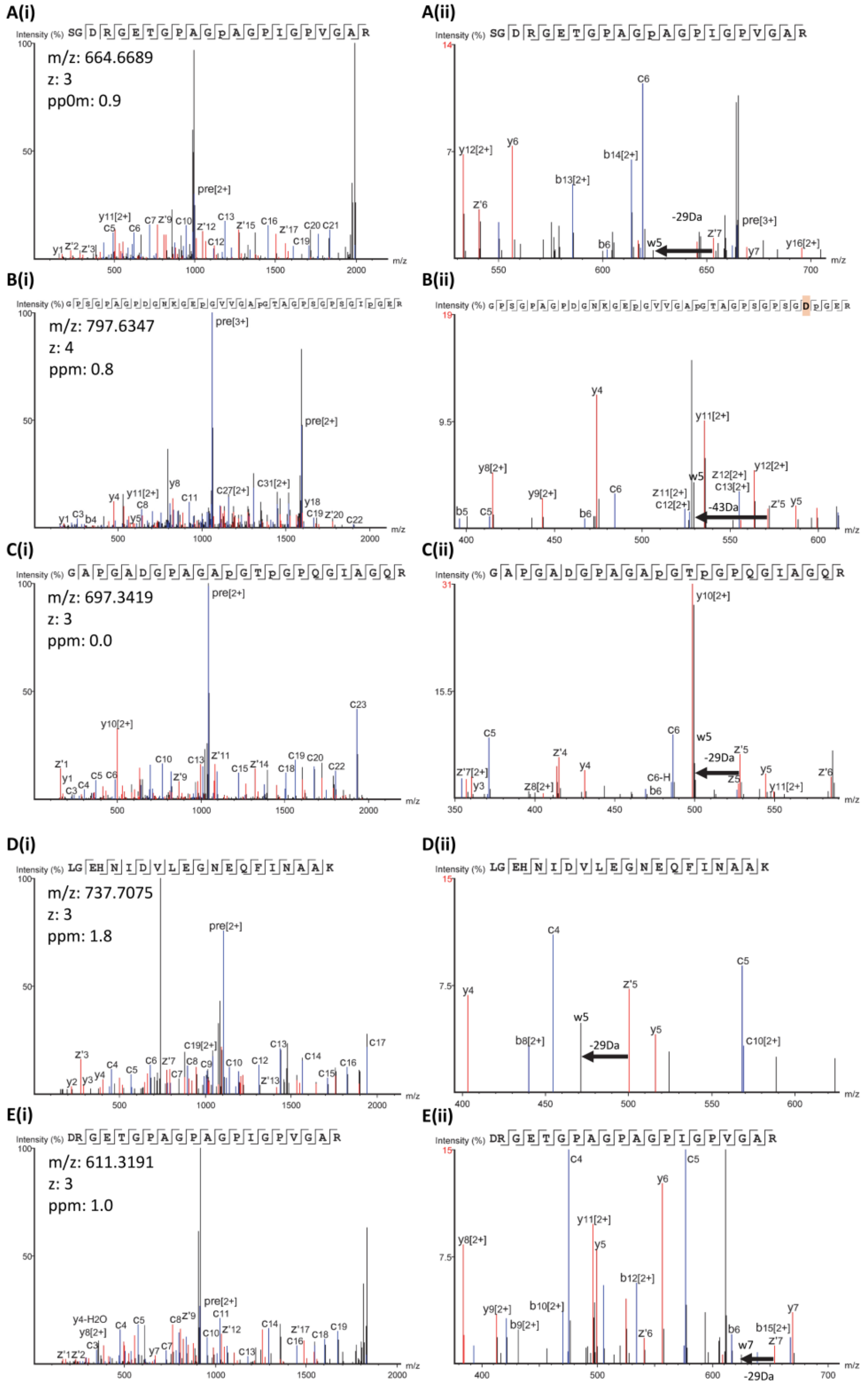

Fig. S4.

A-E(i), peptides in collagen type I from *Hippopotamus amphibius* containing leucine or isoleucine residues, fragmented using EThcD fragmentation (27% supplemental HCD activation), A-E(ii) focus on the parts of the spectra showing the characteristic loss of either 29 or 43 Da, with the presence of the w-ion.

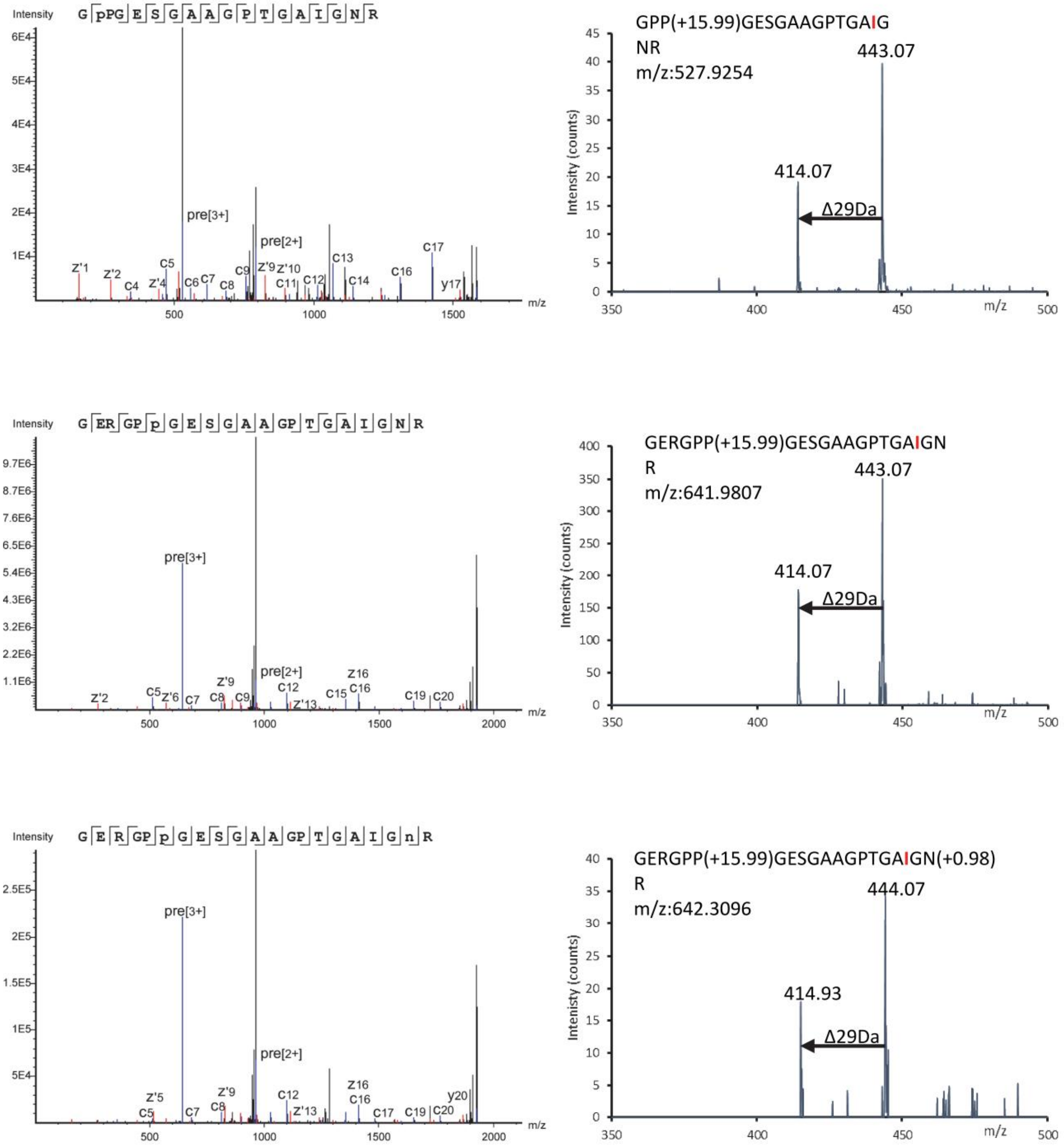

Fig. S5.

From left to right, the ETD-MS2 spectra of the 3+ charge state of the targeted peptide sequence followed by the corresponding MS3 spectra, showing a neutral loss of 29 Da to confirm isoleucine at residue 456 in collagen α2(I) of *Hippopotamus amphibius*.

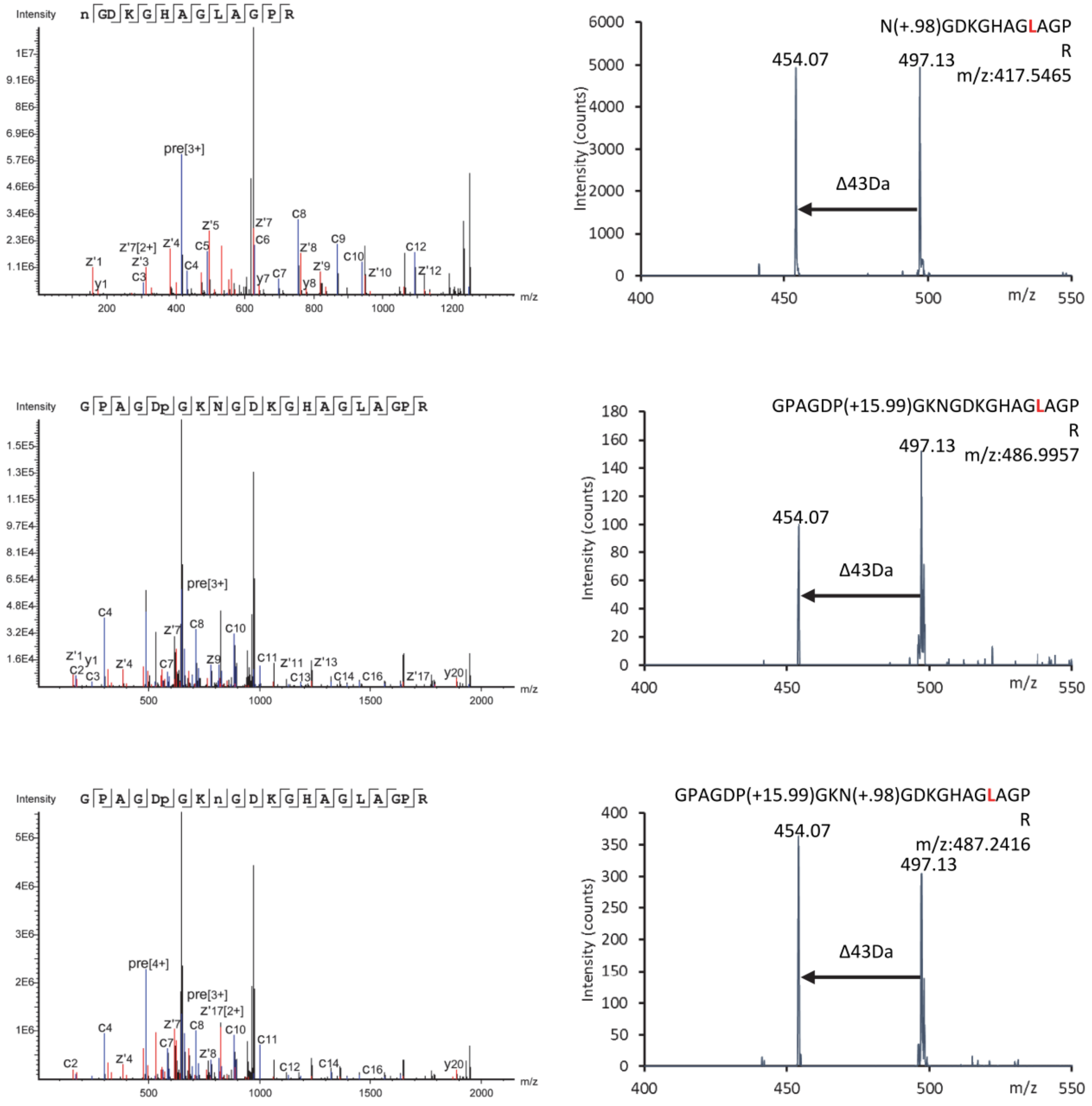

Fig. S6.

From left to right, the ETD-MS2 spectra of the 3+ charge state of the targeted peptide sequence followed by the corresponding MS3 spectra, showing a neutral loss of 43 Da, confirming leucine at residue #515 in collagen α2(I) of *Loxodonta africana*.

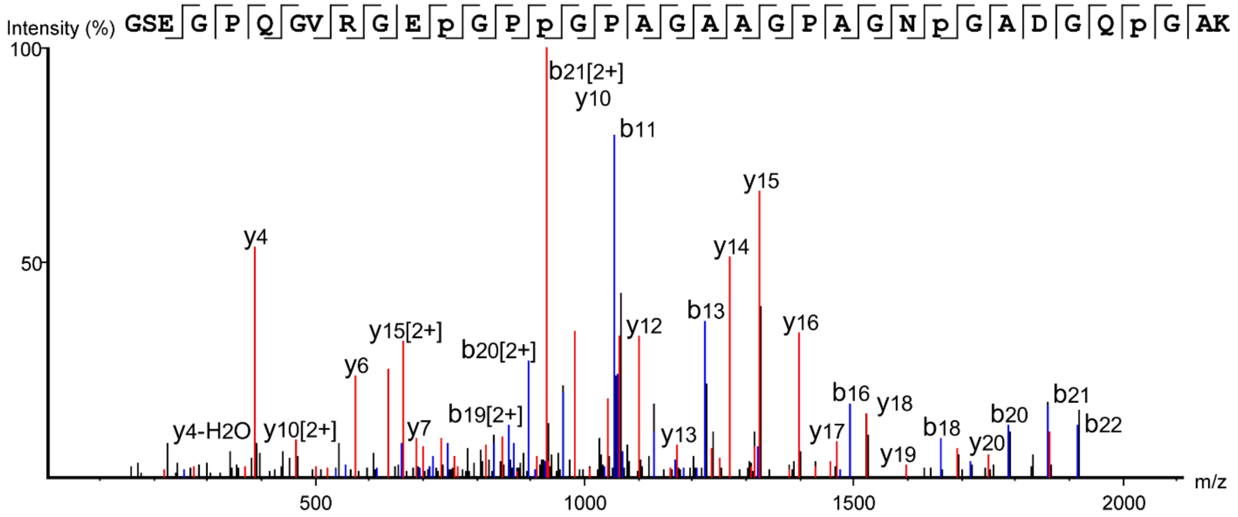

Fig. S7.

Peptide detected in object 12.187.30 that is not species specific, GSEGPQGVRGEP(+15.99)GPP(+15.99)GPAGAAGPAGNP(+15.99)GADGQP(+15.99)GAK, m/z:1061.8286, z:3, ppm:4.1.

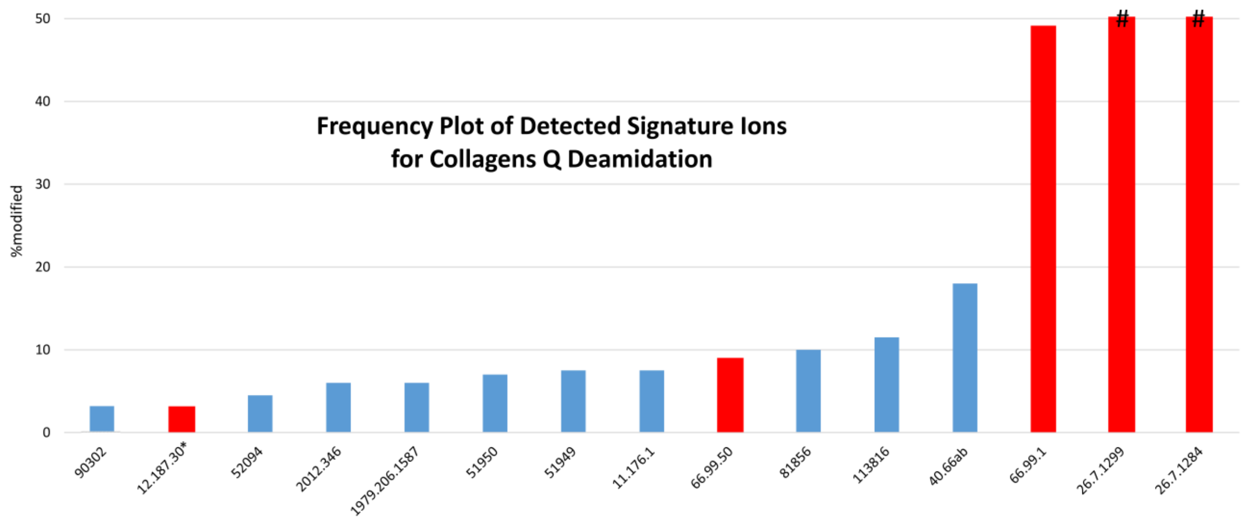

Fig. S8.

Frequency plot of detected signature ions for Q deamidation for collagen alpha-1(I) and collagen alpha-2(I). Blue bars represent samples from 13th to 20th century and red bars represent samples dated from 3900 B.C.-2900 B.C. # means cropped bars, * informs on the low signal-to-noise data used to produce the frequency plot bar. Error bars cannot be provided because the data are resulting from unique injection per sample.

Table S1.
Details of the samples analysed in this research that resulted in identification of collagens type I without taxon identification. The table details ascension number, name of the objects sampled, sampled analyzed, sampling location, notes on condition or treatment, and method used to identify collagens type I.

Table S2.
Effect of the successive sampling procedure performed on exemplar human archaeological bone. Sequence coverages and numbers of peptides identified for collagens α1(I) and α2(I) are shown. Settings used for data processing are described in the Materials and Methods section.

Table S3.
Peptides unique to Bovinae subfamily identified across the three samples, each from a single white key, from the Muselar Virginal (11.176.1, Johannes Ruckers, 1622), using MS/MS sequencing.

Table S4.
Peptides unique to *Physeter microcephalus* identified in the Queen chess piece (2012.346, Scandinavian, 13th c.) using MS/MS sequencing.

Table S5.
Peptides unique to *Physeter microcephalus* identified in sample Hawaii bone pendant (1979.206.1587, 18-19th c.) using MS/MS sequencing.

Table S6.
Peptides unique to Bovinae subfamily identified in sample S2 from the central European saddle decorated with bone plaques (40.66.a,b, ca. 1400–1420) using MS/MS sequencing.

Table S7.
Peptides unique to Cervidae, Elephantidae families and a few other highly unlikely taxa identified in S1 from the central European saddle decorated with bone plaques (40.66.a,b, ca. 1400–1420) using MS/MS sequencing.

Table S8.
Peptides unique to Cervidae, Elephantidae families and a few other highly unlikely taxa identified in S3 from the central European saddle decorated with bone plaques (40.66.a,b, ca. 1400–1420) using MS/MS sequencing.

Table S9.
Sequences coverages and numbers of peptides identified for collagens α1(I) and α2(I) from exemplars in the Mammology collection at AMNH.

Table S10.
Peptides identified using MS/MS sequencing in the three exemplar samples from *Hippopotamus amphibius* where the amino acid substitution from P to A at position 455 of collagen α2(I) was identified.

Table S11.

Peptides identified using MS/MS sequencing in the three exemplar samples from *Loxodonta africana* where the G was identified at residue 326 of collagen α1(I).

Table S12.

Peptides identified using MS/MS sequencing in the three exemplar samples from *Loxodonta africana* where residues 548-551 of collagen α1(I) were confirmed to be GPAG.

Table S13.

Peptides unique to *Hippopotamus amphibius* species identified in Tusk figure of a man (66.99.1, Egypt, ca. 3900–3500 B.C.) using MS/MS sequencing.

Table S14.

Peptides unique to *Hippopotamus amphibius* identified in sample Furniture leg fragment (26.7.1284, Egypt, ca. 2960–2649 B.C.), using MS/MS sequencing.

Table S15.

Peptides unique to Bovinae subfamily identified in Figure of an Asiatic captive (66.99.50, Egypt, ca. 1295–1070 B.C.) using MS/MS sequencing.

Table S16.

Peptides unique to *Hippopotamus amphibius* identified in Figurine or amulet of a hippo on a sled (26.7.1299, Egypt, ca. 2960–2649 B.C.) using MS/MS sequencing.

Table S17.

Examples of peptides unique to *Hippopotamus amphibius* identified in Figurine or amulet of a hippo on a sled (26.7.1299, Egypt, ca. 2960–2649 B.C.) and exemplar sample 113816 identified using a LC-MS-based PMF approach.

Table S18.

Examples of peptides identified in the spoon (12.187.30, Egypt, ca. 3100-2649 B.C.) specific to the *Bos* genus using a LC-MS-based PMF approach.
